# The association between clinical and laboratory findings of bullous pemphigoid and dipeptidyl peptidase-4 inhibitors in the elderly: a retrospective study

**DOI:** 10.3325/cmj.2020.61.93

**Published:** 2020-04

**Authors:** Zrinka Bukvić Mokos, Mikela Petković, Anamaria Balić, Branka Marinović

**Affiliations:** 1 University of Zagreb School of Medicine, Zagreb, Croatia; 2 Department of Dermatovenereology, University Hospital Center Zagreb, Zagreb, Croatia; 3 European Reference Network-Skin Centre, University of Zagreb School of Medicine, Zagreb, Croatia

## Abstract

**Aim:**

To evaluate the association between the use of dipeptidyl peptidase-4 inhibitors (DPP4I) and clinical and laboratory findings of bullous pemphigoid (BP) in patients treated at the European Reference Network – Skin Reference Centre in Croatia.

**Methods:**

This retrospective study enrolled 82 patients treated for BP at the Department of Dermatovenereology, University Hospital Center Zagreb from January 2015 to December 2019. Clinical features of BP, presence of comorbidities, and laboratory findings of anti-BP antibodies and eosinophilia were analyzed in three groups of BP patients: 1) diabetes mellitus (DM) type II patients treated with DPP4I, 2) DM type II patients not treated with DPP4I, and 3) non-DM type II patients.

**Results:**

The average age and anti-BP180 titer were similar in all three groups. DPP4I group had a slightly lower eosinophil level in both peripheral blood (4.89%) and biopsy specimens (87.5%), but the difference was not significant. The prevalence of inflammatory BP in DPP4I group was 76.5%. DPP4I group had significantly higher percentage of patients with chronic renal failure and dementia (52.9% and 11.8%, respectively) compared with non-DPP4I DM (14.3% and 0%, respectively) and non-DM type II patients (15.7% and 0%, respectively).

**Conclusion:**

BP patients treated with DPP4I and those not treated with DPP4Is did not significantly differ in laboratory findings. However, DPP4I treatment was associated with an inflammatory subtype of BP and a higher prevalence of dementia and chronic renal failure. These findings warrant further research into the association of BP and DM with dementia and chronic renal failure.

Bullous pemphigoid (BP) is a skin-directed autoimmune blistering disease, which most commonly affects the elderly during the eighth decade of life (1). The incidence of BP varies from 2.5 to 42.8 cases per million a year and is increasing (1-4). The reason for the increasing incidence is unknown, but it is presumed to be population aging (1,2). In the population older than 80, the incidence is nearly 150-330 cases/million/y (3,5,6), while in the population older than 90 it is 398 cases/million/y among men and 87 new cases/million among women (5,7). Current data show no gender preponderance – some authors found an increased incidence among women and some among men (4,5,7). BP is characterized by the presence of autoantibodies against structural hemidesmosomal proteins BP180 (BPAG 2) and BP230 (BPAG1), which are part of the basement membrane zone (BMZ). Most autoantibodies bind to the extracellular, non-collagenous NC16A domain of BP180, which is the dominant antigenic epitope in BP. Autoantibodies are mostly of IgG type, with predominantly IgG1 and IgG3 subtype, followed by IgG4 subtype. IgA and IgE autoantibodies can also be detected (6). In the early, non-bullous, phase of the disease, IgE antibody exerts its pathogenic role through the well-known mechanism of mast cell degranulation, which clinically presents as urticarial lesions and pruritus (8). High IgE levels were found in 70% of BP patients before starting the therapy and are linked to BP resistance to classical therapeutic options (8,9).

The diagnosis of BP is based on histopathology, direct immunofluorescence (DIF), indirect immunofluorescence, and enzyme-linked immunosorbent assay (ELISA). ELISA for the detection of circulating anti-BP 180 and anti-BP 230 autoantibodies can be used not only for the diagnosis but also for disease activity follow-up. Nowadays, assays for IgE anti-BP antibodies detection are commercially available for ELISA, which opens new possibilities for modern treatment modalities (10).

The etiology of BP is still unclear. Autoreactivity in BP is linked to the genetic factor HLA-DQß1*0301 (11-14). Factors that also participate in the pathogenesis of BP are Th1 and Th2 immune responses, as well as Th17 pathway activation (10). Only in a minority of predisposed individuals, the disease onset is connected to risk factors, such as older age, neurological diseases, viral infections, physical factors (like sunburns or ionizing radiation), and drugs (11-13).

Since BP most commonly affects elderly patients, it is associated with a higher number of comorbidities (cardiovascular diseases, neurological diseases, ie, dementia and Parkinson’s disease, and diabetes mellitus) and a higher mortality rate (2,13-18). Also, the elderly population, owing to multiple comorbidities and malignancies, is exposed to polypharmacy, which can trigger the development of BP.

The relationship between BP and diabetes mellitus (DM) has been well known for decades (14,19,20). DM is one of the most common comorbidities, as well as a significant risk factor of one-year mortality (14). Still, the number of DM patients with BP has been increasing (19,21), and the increase has been attributed to increased use of dipeptidyl peptidase-4-inhibitors (DPP4I or gliptins) (14,22,23). DPP4 is a cell-surface serine exopeptidase, a part of the prolyl oligopeptidase family, found in various tissues (24). In the immune system, DPP4 is found on T lymphocytes and activated NK cells, and it is clustered as CD26 (14,24,25). DPP4 inactivates many proinflammatory cytokines, and the inhibition of DPP4 may be implicated in complex regulation of inflammatory response (25). The exact mechanism of BP development induced by DPP4I is unknown, but there are some drug-dependent differences in clinical and pathological features (14). Published studies showed data inconsistency regarding predominant inflammatory vs non-inflammatory clinical features of BP induced by DPP4I (14,25,26).

DPP4I include vildagliptin, sitagliptin, saxagliptin, linagliptin, and alogliptin. Vildagliptin is most commonly associated with the development of BP, followed by linagliptin (22,23,26-28). Thus, the relationship between BP and those two gliptins was mostly studied, while other drugs from the family have been investigated to a lesser extent.

Since the differences between patients with DPP4I-induced BP and those with DPP4I-non-induced BP are still not clear, the aim of our study was to evaluate the association between the use of DPP4I and clinical and laboratory findings of BP in patients treated at the European Reference Network – Skin Reference Centre in Croatia.

## PATIENTS AND METHODS

This retrospective study enrolled all patients (N = 82) treated for BP from January 2015 to December 2019 at the Department of Dermatovenereology, University Hospital Centre Zagreb. Patients’ records were obtained from the Hospital Information System. Patients were diagnosed with BP according to the International Classification of Diseases, 10th revision, code L12.0.

The definite diagnosis of BP was made by a dermatologist based on clinical findings, histopathology, DIF, and ELISA testing for antibodies against BP 180 and BP 230. For further analyses we used data for anti-BP 180 only as it has higher importance in diagnosis and follow-up than anti-BP 230. The anti-BP 180 (anti-BP 180 NC 16A) antibody titer positivity was defined as ≥20 RU/mL (RU = relative unit).

The collected data included the age at BP onset, sex, anti-BP180 antibodies, the presence of DM type II, chronic renal failure, dementia or neurological disease, and DPP4I treatment before the onset of BP.

We divided BP patients into three groups: 1) DM type II patients treated with DPP4I (DPP4I group); 2) DM type II patients not treated with DPP4I (non-DPP4I group); and 3) non-DM type II patients. Based on clinical data, we distinguished between inflammatory and non-inflammatory BP type. Also, based on diagnostic evaluation, we assessed peripheral eosinophilia and eosinophilia in histopathology specimens to examine if DPP4I-induced BP differed from non-DPP4I induced BP, as shown by other studies (8,26,29). This study was approved by the Ethics Committee of University Hospital Centre Zagreb (02/21A) and was performed in accordance with the principles of the Declaration of Helsinki.

### Statistical analysis

The normality of the distribution of quantitative variables was tested with the Kolmogorov-Smirnov test. The Kruskal-Wallis test was used to assess the difference in the age of onset, peripheral eosinophilia, and anti-BP180 antibody titer between the groups. The Pearson χ^2^ test was used to test the differences in the frequencies of patients older than 65 years at the time of diagnosis, sex, hospitalization, systemic steroid use, eosinophils in the biopsy, positive anti-BP180 antibody, chronic renal failure, dementia, and neurodegenerative diseases between the groups. The level of significance was set at *P* < 0.05. The statistical analysis was performed with TIBCO Statistica 13 (TIBCO Software Inc, Palo Alto, CA, USA).

## RESULTS

DPP4I group included 17 patients (20.7%), non-DPP4I DM group 14 patients (17.1%), and non-DM group 51 patients (62.2%). The groups did not differ in the median age (75.5 years in non-DPP4I, 74.0 in DPP4I group, and 74.0 in the non-DM group; *P* = 0.555). All patients in the DPP4I group were older than 65 years (100%). DPP4I group had a slightly lower level of eosinophils in both peripheral blood (median 2.6%, IQR 1.0-7.0) and biopsy specimens (87.5%) in comparison with the other two groups, but the difference was not significant (*P* = 0.384).

DPP4I group had significantly higher percentage of patients with chronic renal failure and dementia (52.9% and 11.8%, respectively) compared with non-DPP4I DM group (14.3% and 0%, respectively) and non-DM type II patients (15.7% and 0%, respectively) (*P* < 0.005) (Table 1).

**Table 1:** Clinical characteristics of patients with bullous pemphigoid without and with type-2 diabetes mellitus, treated and not treated with dipeptidyl peptidase-4 inhibitors (DPP4I)^‖^

	Non-DPP4I DM patients (n = 14)	DPP4I treated DM patients (n = 17)	Non-DM patients (n = 51)	*p*
Average age of onset in years (median, interquartile range)	75.5 (69-81)	74.0 (71-81)	74.0 (66-80)	0.555*
Older >65 years at the time of diagnosis, n (%)	13 (92.9)	17 (100.0)	45 (88.2)	0.316^†^
Sex ratio (male:female)	7:7	8:9	18:33	0.496^†^
Hospitalization, n (%)	9 (64.3%)	13 (76.5)	35 (68.6)	0.745^†^
Systemic steroid use, n (%)	14 (100.0)	17 (100.0)	51 (100.0)	0.144^†^
Eosinophils in biopsy, n (%)^‡^	10 (90.9)	14 (87.5)	43 (89.6)	0.957^†^
Percent of peripheral eosinophilia (median, interquartile range)	7.4 (2.3-12.2)	2.6 (1.0-7.0)	6.2 (1.4-13.4)	0.384*
Anti-BP180 antibody positive, n (%)^§^	13 (100.0)	15 (93.8)	46 (92.0)	0.573^†^
Anti-BP180 antibody titer (median, interquartile range), U/mL	142.9 (103.9-270.2)	139.9 (45.4-264.2)	168.2 (88.0-266.6)	0.798*
Chronic renal failure, n (%)	2 (14.3)	9 (52.9)	8 (15.7)	0.005^†^
Dementia, n (%)	0 (0.0)	2 (11.8)	0 (0.0)	0.020^†^
Neurological disorders, n (%)	2 (14.3)	4 (23.5)	10 (19.6)	0.811^†^

*Kruskal-Wallis test.

†Pearson χ^2^ test.

‡Data on 7 patients in the non-DM group, 3 patients in the non-DPP4I DM group, and 2 patients in the DPP4I group were missing.

§Data on 5 patients in the non-DM group, 1 patient in the non-DPP4I DM group, and 2 patients in the DPP4I group were missing.

‖All of our groups were heterogeneous with high variability and a small number of participants, so the comparison of each group with another would not contribute to statistical significance.

The median age of onset was 74.0 years. The most commonly used drug was vildagliptin, in 47.06% of patients, and 94.12% of patients concomitantly received another antidiabetic drug. The median period from starting DPP4I therapy to BP onset was 8.27 months. In seven patients, data on treatment duration were lost. In this group, 93.75% of patients had positive BP180 NC16A autoantibody, with a median titer of 139.9 RU/mL (IQR 45.4-264.2 RU/mL). Only 23.5% of DPP41 patients had the non-inflammatory subtype (Table 2).

**Table 2:** Characteristics of elderly diabetic patients treated with dipeptidyl peptidase-4 inhibitors (DPP4I) who developed bullous pemphigoid (BP)

Sex	Age	DPP4I	Period from DPP4I to onset of BP (months)	Concomitant diabetic drugs	Anti-BP180 antibody positive (yes/no)	Anti-BP180/ antibody titer (U/mL)	Eosinophils in skin biopsy (yes/no)	BP subtype (inflammatory or non-inflammatory)
F	72	alogliptin	12	I	yes	131.54	yes	inflammatory
F	81	vildagliptin	ND	I + M	yes	144.35	yes	inflammatory
F	80	vildagliptin	12	M	yes	139.9	yes	inflammatory
F	79	vildagliptin	1	M + S + T	yes	233.54	yes	inflammatory
F	80	vildagliptin	ND	S + T	yes	403.47	no	inflammatory
M	84	sitagliptin	ND	S	yes	353.04	yes	inflammatory
M	87	linagliptin	14	M + S	yes	141.95	ND	non-inflammatory
M	69	vildagliptin	ND	M	yes	45.43	yes	inflammatory
M	73	linagliptin	0	M + S	yes	35.69	no	inflammatory
M	69	linagliptin	12	I	no		yes	non-inflammatory
F	73	vildagliptin	3	M + I	yes	264.24	yes	inflammatory
M	81	vildagliptin + linagliptin	7	M + I	yes	33.19	yes	non-inflammatory
M	74	linagliptin	ND		ND	ND	yes	inflammatory
F	69	vildagliptin	16	I	yes	50.59	yes	inflammatory
F	73	alogliptin	13	S	yes	138.31	yes	non-inflammatory
F	68	sitagliptin	1	M	yes	333.10	yes	inflammatory
M	81	linagliptin	ND	SGLT2-I	yes	24.7	yes	inflammatory

*ND – not determined; M – metformin; I – insulin; S – sulfonylurea; T – thiazolidinedione; SGLT2-I – sodium-glucose transport protein 2 inhibitor.

## DISCUSSION

This study showed a higher prevalence of chronic renal failure and dementia in patients receiving DPP4Is. These results are contradictory to the results of other studies, which showed that DPP4I treatment was associated with a lower incidence of dementia and may have a protective effect on renal disease progression (30,31). Our findings might be explained by a slightly older age in our DPP4I group in comparison to the other two groups and a small number of patients. In addition, older patients in our study might have neglected DM for some time and started treatment at a later stage, which is connected with the development of comorbidities.

DPP4I group in this study had a median age of 74.0 years, which is slightly lower than in other studies (14,22,23,26,32,33). All the patients in this group were older than 65 years. Our results showed a slight, but not significant, preponderance of women, which is in concordance with other findings (27).

The higher incidence of BP in the elderly may be partly explained by an altered immune system. Immunosenescence, defined as age-related changes in the immune system, and its role in the development of autoimmune diseases among the elderly, is an important subject of scientific research (34). Innate and adaptive immune systems change with aging. Besides immunosenescence, there is also cellular senescence, a process connected with the development of some age-related diseases. A distinct phenotype change of the cell is the acquisition of senescence-associated secretion phenotype (SASP), which includes secreted inflammatory, growth-regulating, and remodeling factors. Interestingly, some of the cytokines up-regulated by SASP are the same cytokines involved in the pathomechanism of BP: interleukin (IL)-1, IL-6, and IL-8, tumor necrosis factor α, and matrix metalloproteinases (11,35,36). Inflamm-aging can be defined as low-grade inflammation combined with older age. It is characterized by an increase in pro-inflammatory markers and circulating cytokines and is associated with dementia, atherosclerosis, and DM type II (36). All of these age-related alterations explain why BP is more frequent in the older population but also give us more insight into the connection of BP with different co-morbidities.

The disease is caused by autoantibodies directed against the structural components of the BMZ, particularly BP180, and specifically the anti-NC16A domain, which contains a dominant pathogenic epitope (23). It is still unknown what triggers the development of auto-antibodies against BP180. There are many gaps in the understanding of the etiopathogenesis of the disease, but the association of gliptin therapy with the development of BP has been well confirmed (14,22,23,26,28,32,33,37,38) (Table 3).

**Table 3:** Characteristics of eligible gliptin-associated bullous pemphigoid studies

Study	Country	Cases/controls	Latency time (months)	Age (years)	Sex, male (%)	Mono/multi
Varpuluoma et al (22)	Finland	3397/12941	14.72	76.6	40.3	multicentric
Kawaguchi et al (23)	Japan	32/136	48.4	78.9	46.8	monocentric
Kridin et al (26)	Israel	82/328	/	79.1	55.3	monocentric
						
Benzaquen et al (32)	France	61/122	8.2	79.1	49.2	multicentric
Lee et al (33)	Korea	670/670	/	75.3	51.0	multicentric
Lindgren et al (38)	Finland	10/17	13.4	80.3	40.0	monocentric

DPP4, or CD26, can act anti-inflammatory, by tearing and inactivating proinflammatory cytokines. Hence, DPP4I could promote an inflammatory response by activating cytokines, resulting in tissue damage and blister formation (37). It is known that eosinophils, along with neutrophils, produce serine proteases that damage the BMZ. Some authors showed a sparse infiltration of eosinophils in BP patients treated with DPP4I (28). The exact mechanism is still unclear, but it has been proposed that decreased eotaxin-1 production may act via the inhibition of eosinophil-recruiting cytokines, eg, IL-13, and transforming growth factor-β1 (28). We found a slightly lower level of eosinophils in both peripheral blood (4.89%) and biopsy specimens (87.5%) in the DPP4I group in comparison with the other two groups, but the result was not significant. The majority of DPP4I patients in our study (76.5%) had inflammatory BP clinical subtype. These results are in concordance with the studies that did not find a significant difference between BP patients who were and were not treated with DPP4I (26,38,39).

The combination of DPP4I proinflammatory effect with inflamm-aging may act as a major trigger in BP pathogenesis and its onset. Contrary to the above, it seems that autoantibodies in DPP4I-induced BP are directed against other portions of BP180 antigen besides the NC16A domain (40). A recent study found that more DM patients treated with DPP4I had BP180-full-length and BP180 NC16A ELISAs than patients not treated with DPP4I. Patients with anti-full length BP180 IgG-positive ELISA were significantly older than those with anti-full length BP180 IgG-negative ELISA, which indicates that older age in combination with DPP4I treatment may be a risk factor for the development of anti-full length BP180 IgG antibodies. On the other hand, a higher percentage of anti-full length BP 180 may indicate that DPP4I induce the development of BP by some still unknown mechanism other than the BP180 NC16A epitope spreading (40). Our results showed no significant difference in both percentage of positive anti-BP180 antibodies and titer range between all three groups of patients, which is similar to the results of other studies (26,38,39).

There was no significant difference in hospitalization rate and systemic steroid administration rate between all three groups, which is in concordance with previous studies (23).

A meta-analysis by Phan et al (27) demonstrated a significant association between DPP4I and BP. Among gliptins, vildagliptin was most strongly associated with BP development and had the highest odds ratio, although this DPP4I was least frequently prescribed (22,23,26,27,30,33,41). This can be explained by the fact that vildagliptin is the least selective DPP4I and more strongly inhibits the activity of other DPP isoenzymes, such as DPP8 and DPP9, in a way that is still unknown (23). The usage of other antidiabetic drugs has not been shown to increase the risk for BP, so we did not analyze this factor (42). However, 94.12% of our patients treated with DPP4I received concomitant antidiabetic therapy. Our study showed a mean period of 8.27 months from starting DPP4I treatment to BP onset, which is slightly lower than in other studies (27).

The present study has some limitations, most notably a small number of patients and lack of control group. In conclusion, we found no significant differences in the laboratory findings (including ELISA tests and eosinophil count in skin biopsy and peripheral blood) of BP patients treated with DPP4I and those not treated with DPP4Is. However, DPP4I treatment was associated with an inflammatory subtype of BP and a higher prevalence of dementia and chronic renal failure. This result warrants future studies on the association of BP and DM with dementia and chronic renal failure.
